# Classifying Dementia Diagnoses in the Baltimore Experience Corps Trial Using Medicare and Maryland’s Health Data

**DOI:** 10.1093/geroni/igaf122.3560

**Published:** 2025-12-31

**Authors:** Vishaldeep Sekhon, Qian-Li Xue, Kyle Moored, Halima Amjad, Emily Richards, Eric Slade, George Rebok, Michelle Carlson

**Affiliations:** Johns Hopkins University School of Medicine, Baltimore, Maryland, United States; Johns Hopkins University, Baltimore, Maryland, United States; Johns Hopkins Bloomberg School of Public Health, Baltimore, Maryland, United States; Johns Hopkins University School of Medicine, Baltimore, Maryland, United States; Johns Hopkins Bloomberg School of Public Health, Baltimore, Maryland, United States; Johns Hopkins University School of Nursing, Baltimore, Maryland, United States; Johns Hopkins University, Baltimore, Maryland, United States; Johns Hopkins Bloomberg School of Public Health, Baltimore, Maryland, United States

## Abstract

Centers for Medicare and Medicaid Services (CMS) administrative data are among the most practical sources for understanding the incidence of dementia over time in older populations, but have yet to be widely used in intervention studies. Through the National Institute of Aging’s LINKAGE program, the Baltimore Experience Corps Trial (2006-2022) is one of the first intervention studies to leverage Maryland’s state-level Health Information Exchange (HIE) (2013-2022) data, along with CMS administrative data (2008-2022) to sensitively capture participants’ healthcare utilization. After evaluating multiple established dementia classification algorithms, we used the Bynum evaluation and management (Bynum-EM) algorithm to identify dementia using International Classification of Diseases (ICD-9,10) codes from CMS Fee-for-Service, Medicare Advantage, and HIE data. We further adjudicated dementia diagnosis by its subtypes (Alzheimer’s, mixed/vascular) and incorporated dementia-free mortality. Of 702 participants, 520 were successfully linked with CMS/HIE administrative data. These linked participants were predominantly female (84%), Black (95%), with an average age of 68±6 years and an average of two comorbidities. Over the 14-year follow-up, 26% (n = 135) of the BECT cohort developed dementia, with 8% (n = 32) of them diagnosed with vascular or mixed dementia. About 44% (n = 230) of participants died over time, with 27% having dementia-free mortality. Our study demonstrated the value of both CMS Medicare and state-level HIE data in classifying incident dementia and its subtypes among older adults, providing a useful approach to evaluating long-term intervention effects on dementia risk without requiring in-person follow-up.

